# A Perl toolkit for LIMS development

**DOI:** 10.1186/1751-0473-3-4

**Published:** 2008-03-19

**Authors:** James A Morris, Simon A Gayther, Ian J Jacobs, Christopher Jones

**Affiliations:** 1 Translational Research Laboratories, UCL EGA Institute for Women's Health, University College London, UK

## Abstract

**Background:**

High throughput laboratory techniques generate huge quantities of scientific data. Laboratory Information Management Systems (LIMS) are a necessary requirement, dealing with sample tracking, data storage and data reporting. Commercial LIMS solutions are available, but these can be both costly and overly complex for the task. The development of bespoke LIMS solutions offers a number of advantages, including the flexibility to fulfil all a laboratory's requirements at a fraction of the price of a commercial system. The programming language Perl is a perfect development solution for LIMS applications because of Perl's powerful but simple to use database and web interaction, it is also well known for enabling rapid application development and deployment, and boasts a very active and helpful developer community. The development of an in house LIMS from scratch however can take considerable time and resources, so programming tools that enable the rapid development of LIMS applications are essential but there are currently no LIMS development tools for Perl.

**Results:**

We have developed ArrayPipeline, a Perl toolkit providing object oriented methods that facilitate the rapid development of bespoke LIMS applications. The toolkit includes Perl objects that encapsulate key components of a LIMS, providing methods for creating interactive web pages, interacting with databases, error tracking and reporting, and user and session management. The MT_Plate object provides methods for manipulation and management of microtitre plates, while a given LIMS can be encapsulated by extension of the core modules, providing system specific methods for database interaction and web page management.

**Conclusion:**

This important addition to the Perl developer's library will make the development of in house LIMS applications quicker and easier encouraging laboratories to create bespoke LIMS applications to meet their specific data management requirements.

## Background

High throughput investigation techniques such as microarrays are now well established in scientific research. As the costs of these techniques fall, greater numbers of laboratories are adopting these approaches. High throughput techniques and the equipment associated with them provide researchers with a number of new challenges, one of which is the management and storage of the vast quantities of data they generate. Laboratory notebooks and computer spreadsheets still form the data management strategy for many research scientists, and this simple approach has advantages in the ease of storage and viewing of the data for low throughput approaches however this approach is inadequate for the amounts of data generated using the latest laboratory techniques.

The solution to this data management challenge is the implementation of a Laboratory Information Management System (LIMS), a computer application designed to track samples, store data generated by laboratory equipment and experiments and report these data. There are numerous commercial LIMS applications available however these can be very costly and sometimes overly complex. The system may also fail to support all the requirements a laboratory has, such as for a specialised piece of equipment with unique data import and export formats. For all of these reasons the development of an in house bespoke LIMS is often the best solution for a laboratory's data management requirements.

The programming language Perl [1] has established itself as a standard in the bioinformatics community due to a number of features which make it a perfect solution for developing bioinformatics applications. Perl provides advanced, yet simple to use database support and comprehensive support for internet services such as common gateway interface (CGI) programming. Additionally Perl boasts a very active and helpful developer community which includes the Comprehensive Perl Archive Network (CPAN [2]), a large repository of third party modules containing reusable code solutions for many common development problems. As such Perl is an excellent option for the development of a LIMS.

As the creation of LIMS can take considerable time and resources, tools that can assist developers in common development tasks such as database interfacing and user interface programming are essential. Currently there are no LIMS development tools available for Perl; as a result we have developed the ArrayPipeline Toolkit providing object oriented methods for the rapid deployment of LIMS applications.

## Implementation

ArrayPipeline has been developed as a suite of Perl modules written in an object oriented style to provide reusable code that behaves in a consistent manner. The toolkit includes a number of methods that extend the functionality of two Perl modules; DBI.pm the standard database interface module for Perl and CGI.pm, the common gateway interface class for Perl. Alongside Perl the toolkit has been developed using two other standards in bioinformatics application development; the open source database MySQL [3] and the freely available Apache web server 4]. Although we have chosen to use MySQL and Apache for development of the toolkit it should be possible to use any database based on the Structured Query Language (SQL) and any CGI capable web server.

## Results

### LIMS::Controller

LIMS::Controller is the core component of the ArrayPipeLine toolkit. It provides a single object handle for the multiple sessions and routes of data input and output created by the many tools necessary for building LIMS, such as CGI and DBI libraries, and also handles many basic administration functions. LIMS::Controller inherits from classes within two modules, LIMS::Web::Interface and LIMS::Database::Util, which independently control CGI and DBI services respectively. The methods for database interaction available in LIMS::Database::Util create a fully featured object layer to any relational MySQL database, with the majority of methods compatible with any SQL based database. The methods that make up the database object layer all extend code from Perl DBI (DataBase Interface) library, providing methods for inserting, updating and retrieving database rows. All the available methods are simple and easy to use but still provide flexibility to perform more complex database operations. There is also flexibility in the data structures used for inserting, updating and retrieving data. Other file formats, such as file formats, are handled as binary objects for storage in the database.

The development of interactive web pages for user interaction, using HTML and the common gateway interface (CGI) protocol, is supported by a number of methods in the LIMS::Web::Interface module. The majority of these are extensions of methods from the Perl CGI library. Firstly upon the creation of a new LIMS object a Perl CGI object is automatically created, which can then be used to create web fill-out forms and parse their contents. One challenge associated with developing CGI applications is the maintenance of state across multiple pages. The creation of correctly formatted URLs incorporating CGI parameter values can be complex, so the LIMS::Web::Interface module contains forwarding methods that will transfer data across multiple CGI pages. The use of formatting methods also means that URLs are not hard coded into the CGI script, so any change to a URL can be made to a single configuration file rather than to multiple CGI scripts.

The Perl language and the CGI and DBI libraries all have extensive error capture and reporting capabilities. The modules described above both contain error handling methods that, *via *LIMS::Controller, provide a single consistent interface for capturing and reporting errors from Perl, CGI and DBI. One significant extension to the DBI library code is in the case of database inserts and updates; upon the generation of any errors, the LIMS object will issue a rollback command cancelling any changes made to the database during the transaction. The objects 'kill' method can test if any errors have been captured, and if so kill the script and print out the errors in either plain text or formatted HTML.

Key to a successful LIMS is maintenance of data integrity, and this can be achieved through restricting access to sensitive pages to known and trusted users. The LIMS::Controller module extends Apache and MySQL authorization and security by providing methods for verifying the username and password of anyone attempting to access a restricted web page, using a *'user_information' *table in the LIMS database. If this step is successful then a session is automatically generated, however if inactive for longer than the set session time the session expires and authentication is again required. A *'log_out' *method is also provided for ending the current session.

### Sample tracking and management

One of the key components of high-throughput technologies is the microtiter plate, used for storage and manipulation of experimental samples. The LIMS::MT_Plate module encapsulates a microtitre plate, providing methods for creating and manipulating microtitre plate objects of different formats including 96, 384 and 1536 well plates, as well as individual tubes. A plate object for each microtitre format can be created, as child classes of the parental plate class they inherit all standard methods as well as specific information pertaining to each format. The standard features which can be used by all plate objects include methods for filling multiple and individual wells, the identity of samples in plate can be returned either as individual wells or collectively. Some of the most useful features of the plate object are concerned with whole plate manipulations, and methods are available to perform plate to plate transfers, join plates of a similar format into a larger format, or combine plates. The LIMS::MT_Plate module contains further methods for dealing with plates and samples, and is extremely useful for developing applications for tracking and manipulating samples contained in microtitre plates. This is a standalone module, which can also inherit from the LIMS package to automate data input and export.

### LIMS object

The LIMS object encapsulates a given LIMS, it inherits from LIMS::Controller, and enables simplified interaction with all the components of the LIMS including the database, web pages and plates. The object can quickly and easily be setup to work with a specific LIMS through editing a configuration file with parameters appropriate to the system in question. The configuration file parameters include database connection details, web set-up, directory structure and sections of HTML code for common web elements such as the page headers, footers and menus.

Once configured, the LIMS object will significantly increase the speed of development of applications for the associated LIMS, such as in the creation of new web pages. Each web page is divided into a header, sidebar and footer, the HTML for which is stored in the configuration file. The generation of the LIMS object includes user session control utilising form parameters, and provides methods for formatting the HTML layout of a new page quickly and easily; the header and sidebar are both printed using a single method call, the page content is then added, and finally the page footer and parameter forwarding is printed with another method call.

### LIMS::ArrayPipeLine

LIMS::ArrayPipeLine is an example of a LIMS object, and is the module that encapsulates our own laboratory's microarray LIMS. Together with a database schema, configuration file and cgi scripts, this module serves as a suitable template for most LIMS requirements, including the manipulation of microtitre plates, recording procedure and protocol information, and tracking individual protocol components. Access to LIMS::Controller functions is provided by a single class, LIMS::ArrayPipeLine::Pipeline_Owner, which enables the simple creation of inheriting classes that can interact with the LIMS database. An example of this is the module LIMS::ArrayPipeLine::Pipeline_Plate, which also inherits from LIMS::MT_Plate and provides methods that enable the creation of MT_Plate objects from database entries and vice versa.

LIMS::Sample is a 'stripped down' version of LIMS::ArrayPipeLine, and is provided in the LIMS::Controller distribution as an example system. It consists of the components required for the tracking of microtitre plates and their contents, and includes a database schema, configuration file, CGI scripts and detailed installation instructions. Detailed annotation of the CGI scripts in this example system help to explain many of the features of the LIMS::Controller toolkit.

## Conclusion

The LIMS toolkit provides tools for the rapid development of LIMS applications including methods for the automation of web page development tasks, database interaction and for dealing with microtitre plates. These tools combine to provide the efficient creation of web pages for user interaction, allowing the developer to concentrate on functionality rather than layout. Methods for the creation and manipulation of microtitre plate objects importantly provide a consistent way for dealing with experimental samples contained in plates or tubes. The methods for database interaction provide a simple and consistent interface to any SQL based database for routine tasks such as inserts, updates and selects. The toolkit also provides flexibility in the methods, allowing more complex database operations to be performed. This provides consistency of design and purpose, while reducing the risk of errors in the code.

This important addition to the Perl developer's library will facilitate the rapid development of bespoke LIMS applications using Perl.

## Availability and requirements

**Project name**: ArrayPipeline

**Project home page**: 

**Operating systems(s)**: Platform independent

**Programming Language**: Perl

**Other Requirements**: Perl 5 or higher

**License**: GNU General Public License (GPL)

**Restrictions**: None

## Competing interests

The author(s) declare that they have no competing interests.

## Authors' contributions

CJ led ArrayPipeline project development. JAM and CJ wrote all of the ArrayPipeline code and prepared this manuscript. IJJ and SAG provided infrastructure and funding support. SAG is the Director of Research at the Translational Research Laboratories. All authors have read and approve the final manuscript.
